# Supplementary material for: Protocol for a scoping review of multi-omic analysis for rare diseases
Source: BMJ Open. 2019 May 5;9(5):e026278. doi: 10.1136/bmjopen-2018-026278 (PMC6501961; doi:10.1136/bmjopen-2018-026278)
Supplement: Supplementary file 1 [file bmjopen-2018-026278supp001.pdf]

Supplementary materials and methods.

Protocol for a scoping review of multi-omic analysis for rare diseases.

Ms Katie Kerr, Dr Helen McAneney, Dr Amy Jayne McKnight.

Table S1 - Search terms for Ovid MEDLINE(R) ALL.

#	**Searches**
1	Rare Diseases/
2	("rare disease*" or "rare cancer*" or "rare syndrome*" or "rare disorder*").mp.
3	multi?omic*.mp.
4	genomics/ or epigenomics/ or proteomics/ or transcriptomics/ or metabolomics/
5	"integrat* omic*".mp. [mp=title, abstract, original title, name of substance word, subject heading word, floating sub-heading word, keyword heading word, protocol supplementary concept word, rare disease supplementary concept word, unique identifier, synonyms]
6	"integrat* analys*".mp. [mp=title, abstract, original title, name of substance word, subject heading word, floating sub-heading word, keyword heading word, protocol supplementary concept word, rare disease supplementary concept word, unique identifier, synonyms]
7	1 or 2
8	3 or 4 or 5 or 6
9	7 and 8

Table S2 – Search terms for Embase via Ovid.

#	**Searches**
1	rare disease/
2	("rare disease*" or "rare cancer*" or "rare syndrome*" or "rare disorder*").mp.
3	multi?omic*.mp.
4	genomics/
5	epigenetics/
6	proteomics/
7	transcriptomics/
8	metabolomics/
9	epigenomic*.mp. [mp=title, abstract, heading word, drug trade name, original title, device manufacturer, drug manufacturer, device trade name, keyword, floating subheading word, candidate term word]
10	"integrat* omic*".mp.
11	"integrat* analys*".mp.
12	1 or 2
13	3 or 4 or 5 or 6 or 7 or 8 or 9 or 10 or 11
14	11 and 12

Table S3. Template for customised data extraction form.

**First author and publication year**	**Title and digital object identifier**	**Reference list number**	**Research objective**	**Phenotypes reported**	**Experimental controls used**	**Statistical analysis**	**Study design and participant information**	**Key results**
