# Supplementary material for: Protocol for a scoping review of multi-omic analysis for rare diseases
Source: BMJ Open. 2019 May 5;9(5):e026278. doi: 10.1136/bmjopen-2018-026278 (PMC6501961; doi:10.1136/bmjopen-2018-026278)
Supplement: Author's manuscript [file bmjopen-2018-026278.draft_revisions.pdf]

BMJ Open is committed to open peer review. As part of this commitment we make the peer review history of every article we publish publicly available.

When an article is published we post the peer reviewers' comments and the authors' responses online. We also post the versions of the paper that were used during peer review. These are the versions that the peer review comments apply to.

The versions of the paper that follow are the versions that were submitted during the peer review process. They are not the versions of record or the final published versions. They should not be cited or distributed as the published version of this manuscript.

BMJ Open is an open access journal and the full, final, typeset and author-corrected version of record of the manuscript is available on our site with no access controls, subscription charges or pay-per-view fees (http://bmjopen.bmj.com).

If you have any questions on BMJ Open's open peer review process please email info.bmjopen@bmj.com

BMJ Open

Protocol for a scoping review of multi-omic analysis for rare diseases.

Journal:	*BMJ Open*
Manuscript ID	bmjopen-2018-026278
Article Type:	Protocol
Date Submitted by the Author:	24-Aug-2018
Complete List of Authors:	Kerr, Katie; Queen's University Belfast, Regional Genetics Centre; Queen's University Belfast Centre for Public Health, Institute of Clinical Sciences<br>McAneney, Helen; Queen's University Belfast School of Medicine Dentistry and Biomedical Sciences, Centre for Public Health<br>McKnight, Amy; Queen's University Belfast, Centre for Public Health
Keywords:	Rare disease, Multi-omics, Biomarker, Genomics, Epigenomics

SCHOLARONE™
Manuscripts

For peer review only

Title:

Protocol for a scoping review of multi-omic analysis for rare diseases.

Authors:

Ms Katie Kerr (BSc), Dr Helen McAneney (PhD), Dr Amy Jayne McKnight (PhD).

Author affiliations:

Centre for Public Health, Queen's University Belfast, Northern Ireland.

Mailing address:

Dr. Amy Jayne McKnight (corresponding author)

c/o Regional Genetics Centre

Level A, Tower Block, Belfast City Hospital

Lisburn Road, BT9 7AB

Phone: +44 (0)28 9063 8460

Email: a.j.mcknight@qub.ac.uk

Word count: 1395

For peer review only

For peer review only - http://bmjopen.bmj.com/site/about/guidelines.xhtml

ABSTRACT

Introduction: The development of next generation sequencing technology has enabled cost-efficient, large scale, multiple 'omic' analysis, including epigenomic, genomic, metabolomic, phenomic, proteomic and transcriptomic research. These integrated approaches hold significant promise for rare disease research, with the potential to aid biomarker discovery, improve our understanding of disease pathogenesis and identify novel therapeutic targets. In this paper we outline a systematic approach for a scoping review designed to evaluate what primary research has been performed to date on multi-omics and rare disease.

Methods and analysis: This protocol was designed using the Joanna Briggs Institute methodology for scoping reviews. Databases to be searched will include: MEDLINE, EMBASE, PubMed, Web of Science, Scopus, and Cochrane Database of Systematic Reviews for primary studies relevant to the key terms 'multi-omics' and 'rare disease'. Grey literature databases GreyLit and OpenGrey will also be searched, as well as reverse citation screening of relevant articles and forward citation searching using Web of Science Cited Reference Search Tool. Data extraction will be performed using customised forms and a narrative synthesis of the results will be presented.

Ethics and dissemination: As a secondary analysis study with no primary data generated, this scoping review does not require ethical approval. We anticipate this review will highlight a gap in rare disease research and provide direction for novel research. The completed review will be submitted for publication in peer-reviewed journals and presented at relevant conferences discussing rare disease research and/or molecular strategies.

Keywords

Biomarker, epigenomic, genomic, multi-omic, rare disease.

Article Summary

Strengths and limitations of this study:

For peer review only - http://bmjopen.bmj.com/site/about/guidelines.xhtml

- The proposed scoping review will evaluate primary research that has been conducted using multi-omic approaches for rare disease.
- The data extracted will include the type of 'omic' research, study design, methodological rigour of the studies and outcomes of interest.
- As it would be impractical to attempt to list every rare disease as an individual search term, this review will be limited to research which explicitly specifies in the article that the disease of focus is a rare disease.
- Rare diseases named in this approach will be checked for prevalence to ensure they meet the European Union definition of a rare disease.

For peer review only

INTRODUCTION

Individual rare diseases are defined as affecting less than 5 in 10,000 people in Europe and less than 200,000 people in America at any given time (7.5 in 10,000).[1,2] There are estimated to be between 5,000 and 8,000 different types of rare diseases and cumulatively these affect approximately 350,000,000 people worldwide.[2] Due to the clinical heterogeneity of rare diseases, diagnosis is often difficult and treatment options limited. Rare conditions are often severely debilitating and/or life limiting, with 30% of patients dying before they reach their fifth birthday.[3] Furthermore, approximately 80% of rare conditions have a genetic cause, such as those resulting from a single mutation, polygenic inheritance, or structural chromosome abnormalities.[4]

The development of massively parallel sequencing technologies in the past decade, compared to traditional sequencing methods such as Sanger sequencing, has improved the accuracy and speed of rare disease diagnosis.[5] Much of this next generation sequencing research has focused on genomic analysis, such as The 100,000 Genomes Project, which is a national project based in the United Kingdom attempting to sequence the genomes of people with rare diseases (including some cancers) and their family members. There is a need to build on these genomic resources by utilising integrated multi-omic analysis, a biological approach to research that takes into consideration multiple omic terms, of which there are over 500.[6] These include: epigenomic, genomic, metabolomic, phenomic, proteomic and transcriptomic research.

For peer review only - http://bmjopen.bmj.com/site/about/guidelines.xhtml

While the majority of rare disease studies have improved diagnosis through genomic analysis, other omic strategies such as epigenomics and transcriptomics have been employed. In recent studies, differential methylation has been identified as a potential marker of rare renal diseases such as IgA Nephropathy, and ADPKD [7,8] and rare ophthalmic diseases such as retinitis pigmentosa and Fuchs endothelial corneal dystrophy.[9,10] Additionally, transcriptomic analysis of retinal disease may aid in diagnosis and identifying new treatments.[11] Combining singular omic analysis with other analyses to form an allied multi-omic approach could provide new insights into rare disease molecular profiles, aid in diagnosis, prognosis prediction, and identifying novel treatment targets.

A major challenge facing researchers is interpretation of the large amounts of data generated from next generation approaches. In contrast to common complex diseases, rare disease researchers are also faced with the challenge of generating reliable, high quality data, such as a randomised controlled trial, when they often have an extremely small sample size. To tackle this, international collaboration of research is crucial to include participants of a rare disease who may live in different countries, or even continents. Additionally, there is a need to develop a standardised reporting structure for rare disease omic analysis, such as that which exists for genome wide association studies.[12]

Review aim and objectives.

This scoping review will aim to systematically summarise what research has been conducted into multi-omics and rare disease by:

- Evaluating what primary research studies exist pertaining to multi-omics and rare disease and which type of omic analysis was undertaken.
- Highlighting research outcomes with implications for rare disease diagnosis, treatment or improved understanding of disease mechanisms.

METHODS

For peer review only - http://bmjopen.bmj.com/site/about/guidelines.xhtml

This scoping review protocol was designed using the Joanna Briggs Institute Reviewers Manual 2015 – methodology for scoping reviews.[13]

Eligibility criteria

The inclusion criteria were developed using the PCC (population, concept, context) framework of the Joanna Briggs Institute (JBI) scoping review methodology, with a deliberately wide scope to capture as many relevant research studies as possible. The “population” of this scoping review will be rare diseases meeting the European definition of less than 1 in 2,000.[2] Participants may be rare disease patients of all ages, ethnicity and gender. The “concept” of the review is multi-omics and therefore must include at least two types of omic analysis. The most common omic terms we would expect to see are epigenomics, genomics, metabolomics, phenomics, proteomics and transcriptomics, however any omic term may be included. A comprehensive list of omic terms is available for reference on www.omics.org.[6] The review “context” will be within a primary research study written in English and published prior to the 30th September 2018 and as such other reviews of the literature will not be included in the final synthesis but may be helpful for identification of further relevant studies through reverse screening and forward citation searching.

Information sources and search terms

Articles will be identified primarily by searching the electronic databases MEDLINE, EMBASE, PubMed, Web of Science, Scopus, and Cochrane Database of Systematic Reviews. This large selection of databases will be included in the strategy to perform as wide a search as possible. The search strategy is summarised in Table S1, including search terms which are primarily created for use in MEDLINE and EMBASE, then adapted for the other databases. These terms were devised based on the PCC framework discussed above. In addition to traditional searching, reverse citation screening of the reference lists of relevant articles (i.e. including the key terms such as multi-omics and rare disease) and forward citations (articles which have cited the identified papers) will be searched for using the Web of Science Cited Reference Search tool. The search will be performed in duplicate by two independent personnel to ensure reproducibility, and any discrepancies will be considered by third reviewer.

For peer review only - http://bmjopen.bmj.com/site/about/guidelines.xhtml

Selection of studies, data extraction and analysis

The screening process to select studies will begin with compilation of articles identified from the several databases using reference management software (EndNote X8) and subsequent auto-removal of duplicates followed by additional manual checking and removal of further duplicates missed by the software. Title, keyword and abstract screening will identify any studies which noticeably do not meet the study inclusion criteria, i.e. no discussion of multi-omics or singularly focusing on a common disease. Following this, any papers which initially appear to meet the inclusion criteria will be screened *via* reading the full text of the article. Data will then be extracted from the articles which are selected to be included in the review using a customised data extraction form, (Table S2), to include information about study design, participant information, experimental methods and statistical analysis. This information will be summarised in a table. Critical appraisal of these studies will also be performed using customised forms, two examples of which for are available in Table S3 and S4. The templates are designed for case-control and case report studies, as these are two common study designs often used in rare disease research (there is often too few individuals with a particular rare condition to conduct a randomised controlled trial). A narrative synthesis of the results of this review will be presented.

ETHICS AND DISSEMINATION

As this is a review of previously published data and no primary data will be collected, formal ethical committee approval is not required for this scoping review. This scoping review will highlight primary studies that have been conducted for multi-omic analysis of rare disease and discuss the quality of any such research. Multi-omic analysis of rare diseases has important implications for diagnostic/prognostic biomarker development, identifying new treatments, and improving our understanding of the diseases themselves. Subsequently, any gaps in the literature which are identified will be presented at conferences, research group meetings and submitted for publication in a peer reviewed journal. This review will also form part of a chapter in KK's PhD thesis. This review may help guide future rare disease research.

For peer review only - http://bmjopen.bmj.com/site/about/guidelines.xhtml

AUTHOR CONTRIBUTIONS

AJ McK, HMcA conceptualised the review protocol, KK drafted the review protocol and all authors were involved in preparing and agreeing the final protocol manuscript.

FUNDING STATEMENT

KK is supported by a Department for the Economy Co-operative Awards in Science and Technology (DfE-CAST) studentship award. Funding support was provided by the Medical Research Council – Northern Ireland Executive support of the Northern Ireland Genomic Medicine Centre though Belfast Health and Social Care Trust.

COMPETING INTEREST STATEMENT

No competing interests to declare.

REFERENCES

1. RARE DISEASES - A major unmet medical need. Luxembourg: European Commission 2017.

2. Global Genes. RARE Diseases: Facts and Statistics [Available from: https://globalgenes.org/rare-diseases-facts-statistics/ accessed 09/11/2018.

3. EURODIS. About Rare Diseases [Available from: https://www.eurordis.org/about-rare-diseases accessed 15/03/2018.

4. What is a rare disease? : Genetic Alliance UK; 2016 [Available from: http://www.raredisease.org.uk/what-is-a-rare-disease/ accessed 16/04/2018.

5. Boycott KM, Vanstone MR, Bulman DE, et al. Rare-disease genetics in the era of next-generation sequencing: discovery to translation. *Nature reviews Genetics* 2013;14(10):681-91. doi: 10.1038/nrg3555 [published Online First: 2013/09/04]

For peer review only - http://bmjopen.bmj.com/site/about/guidelines.xhtml

6. Alphabetically ordered list of omes and omics 2016 [Available from: http://omics.org/index.php/Alphabetically_ordered_list_of_omes_and_omics accessed 16/03/2018.

7. Sallustio F, Serino G, Cox SN, et al. Aberrantly methylated DNA regions lead to low activation of CD4+ T-cells in IgA nephropathy. *Clinical science (London, England : 1979)* 2016;130(9):733-46. doi: https://dx.doi.org/10.1042/CS20150711

8. Woo YM, Bae J-B, Oh Y-H, et al. Genome-wide methylation profiling of ADPKD identified epigenetically regulated genes associated with renal cyst development. *Human genetics* 2014;133(3):281-97. doi: https://dx.doi.org/10.1007/s00439-013-1378-0

9. Khuc E, Bainer R, Wolf M, et al. Comprehensive characterization of DNA methylation changes in Fuchs endothelial corneal dystrophy. *PLoS One* 2017;12(4):e0175112. doi: 10.1371/journal.pone.0175112 [published Online First: 2017/04/07]

10. Farinelli P, Perera A, Arango-Gonzalez B, et al. DNA methylation and differential gene regulation in photoreceptor cell death. *Cell Death Dis* 2014;5:e1558. doi: 10.1038/cddis.2014.512 [published Online First: 2014/12/06]

11. Farkas MH, Au ED, Sousa ME, et al. RNA-Seq: Improving Our Understanding of Retinal Biology and Disease. *Cold Spring Harbor Perspectives in Medicine* 2015;5(9):a017152. doi: 10.1101/cshperspect.a017152

12. Little J, Higgins JP, Ioannidis JP, et al. STrengthening the REporting of Genetic Association Studies (STREGA)--an extension of the STROBE statement. *Genetic epidemiology* 2009;33(7):581-98. doi: 10.1002/gepi.20410 [published Online First: 2009/03/12]

13. Joanna Briggs Institute Reviewers' Manual: 2015 edition/supplement. Australia: The Joanna Briggs Institute, 2015.

For peer review only

For peer review only - http://bmjopen.bmj.com/site/about/guidelines.xhtml

Supplementary materials and methods.

Protocol for a scoping review of multi-omic analysis for rare diseases.

Ms Katie Kerr, Dr Helen McAneney, Dr Amy Jayne McKnight.

Table S1. Search strategy for primary use in MEDLINE and EMBASE, adapted for other databases.

#	**Search terms.**
1	(rare disease* OR rare cancer* OR rare syndrome* OR rare disorder*).mp.
2	(multiomic* OR multi-omic*).mp.
3	(integrated omic* OR integrative omic* OR integrated analysis OR integrative analysis).mp.

For peer review only

For peer review only - http://bmjopen.bmj.com/site/about/guidelines.xhtml

Table S2. Template for customised data extraction form.

First author and publication year	Title and digital object identifier	Reference list number	Research objective	Design information	Key results

Abbreviations

For peer review only

Table S3. Template quality appraisal form for case control studies.

		Yes	No	Unclear	Not Applicable	Comments
**1**	Was the primary focus of the paper methylation?					
**2**	Were the groups comparable other than the presence of disease in cases or the absence of disease in controls?					
**3**	Were cases and controls matched appropriately?					
**4**	Was methylation measured in in a standard reliable way?					
**5**	Was methylation measured in the same way for cases and controls?					
**6**	Were confounding factors identified?					
**7**	Were strategies to deal with confounding factors identified?					
**8**	Was the exposure period of interest long enough to be meaningful?					
**9**	What statistical analysis was used and was this analysis appropriate?					
**10**	Were experimental controls used?					

For peer review only

For peer review only - http://bmjopen.bmj.com/site/about/guidelines.xhtml

Table S4. Template quality appraisal form for case report studies.

		Yes	No	Unclear	Not Applicable	Comments
**1**	Was the primary focus of the paper methylation?					
**2**	Was the patient's history described clearly?					
**3**	Was the patient's current clinical condition described clearly?					
**4**	Was methylation measured in in a standard reliable way?					
**5**	Were confounding factors identified?					
**6**	Were strategies to deal with confounding factors identified?					
**7**	Were experimental controls used?					

For peer review only

For peer review only - http://bmjopen.bmj.com/site/about/guidelines.xhtml

BMJ Open

Protocol for a scoping review of multi-omic analysis for rare diseases.

Journal:	*BMJ Open*
Manuscript ID	bmjopen-2018-026278.R1
Article Type:	Protocol
Date Submitted by the Author:	22-Jan-2019

Complete List of Authors:	Kerr, Katie; Queen's University Belfast, Regional Genetics Centre; Queen's University Belfast Centre for Public Health, Institute of Clinical Sciences<br>McAneney, Helen; Queen's University Belfast School of Medicine Dentistry and Biomedical Sciences, Centre for Public Health<br>McKnight, Amy; Queen's University Belfast, Centre for Public Health

**Primary Subject Heading**:	Public health
Secondary Subject Heading:	Genetics and genomics
Keywords:	Rare disease, Multi-omics, Biomarker, Genomics, Epigenomics, Transcriptomics

SCHOLARONE™
Manuscripts

Title:

Protocol for a scoping review of multi-omic analysis for rare diseases.

Authors:

Ms Katie Kerr (BSc), Dr Helen McAneney (PhD), Dr Amy Jayne McKnight (PhD).

Author affiliations:

Centre for Public Health, Queen's University Belfast, Northern Ireland.

Mailing address:

Dr. Amy Jayne McKnight (corresponding author)

c/o Regional Genetics Centre

Level A, Tower Block, Belfast City Hospital

Lisburn Road, BT9 7AB

Phone: +44 (0)28 9063 8460

Email: a.j.mcknight@qub.ac.uk

Word count: 1545

For peer review only

ABSTRACT

Introduction: The development of next generation sequencing technology has enabled cost-efficient, large scale, multiple 'omic' analysis, including epigenomic, genomic, metabolomic, phenomic, proteomic and transcriptomic research. These integrated approaches hold significant promise for rare disease research, with the potential to aid biomarker discovery, improve our understanding of disease pathogenesis and identify novel therapeutic targets. In this paper we outline a systematic approach for a scoping review designed to evaluate what primary research has been performed to date on multi-omics and rare disease.

Methods and analysis: This protocol was designed using the Joanna Briggs Institute methodology for scoping reviews. Databases to be searched will include: MEDLINE, EMBASE, PubMed, Web of Science, Scopus, and Google Scholar for primary studies relevant to the key terms 'multi-omics' and 'rare disease', published prior to 30th December 2018. Grey literature databases GreyLit and OpenGrey will also be searched, as well as reverse citation screening of relevant articles and forward citation searching using Web of Science Cited Reference Search Tool. Data extraction will be performed using customised forms and a narrative synthesis of the results will be presented.

Ethics and dissemination: As a secondary analysis study with no primary data generated, this scoping review does not require ethical approval. We anticipate this review will highlight a gap in rare disease research and provide direction for novel research. The completed review will be submitted for publication in peer-reviewed journals and presented at relevant conferences discussing rare disease research and/or molecular strategies.

Keywords

Biomarker, epigenomic, genomic, multi-omic, rare disease.

Article Summary

Strengths and limitations of this study:

For peer review only - http://bmjopen.bmj.com/site/about/guidelines.xhtml

- The proposed scoping review will evaluate primary research that has been conducted using multi-omic approaches for rare disease.
- The data extracted will include the type of 'omic' research, study design, methodological rigour of the studies and outcomes of interest.
- As it would be impractical to attempt to list every rare disease as an individual search term, this review will be limited to research which explicitly specifies in the article that the disease of focus is a rare disease.
- Rare diseases named in this approach will be checked for prevalence to ensure they meet the European Union definition of a rare disease.

For peer review only

INTRODUCTION

Individual rare diseases are defined as affecting less than 5 in 10,000 people in Europe and less than 200,000 people in America at any given time (7.5 in 10,000).[1,2] There are estimated to be between 5,000 and 8,000 different types of rare diseases and cumulatively these affect approximately 350,000,000 people worldwide.[2] Due to the clinical heterogeneity of rare diseases, diagnosis is often difficult and treatment options limited. Rare conditions are often severely debilitating and/or life limiting, with 30% of patients dying before they reach their fifth birthday.[3] Furthermore, approximately 80% of rare conditions have a genetic cause, such as those resulting from a single mutation, polygenic inheritance, or structural chromosome abnormalities.[4]

The development of massively parallel sequencing technologies in the past decade, compared to traditional sequencing methods such as Sanger sequencing, has improved the accuracy and speed of rare disease diagnosis.[5] Much of this next generation sequencing research has focused on genomic analysis, such as The 100,000 Genomes Project; a national project based in the United Kingdom attempting to sequence the genomes of people with rare diseases (including some cancers) and their family members. There is a need to build on these genomic resources by utilising integrated multi-omic analysis, as is increasingly employed for common complex diseases,[6] a biological approach to research that takes into consideration multiple omic terms, of which there are over 500.[7] These include: epigenomic, genomic, metabolomic, phenomic, proteomic and transcriptomic research.

For peer review only - http://bmjopen.bmj.com/site/about/guidelines.xhtml

While the majority of rare disease studies have improved diagnosis through genomic analysis, other omic strategies such as epigenomics and transcriptomics have been employed. In recent studies, differential methylation has been identified as a potential marker of rare renal diseases such as IgA Nephropathy, and ADPKD [8,9] and rare ophthalmic diseases such as retinitis pigmentosa and Fuchs endothelial corneal dystrophy.[10,11] Additionally, transcriptomic analysis of retinal disease may aid in diagnosis and identifying new treatments.[12] Combining singular omic analysis with other analyses to form an allied multi-omic approach could provide new insights into rare disease molecular profiles, aid in diagnosis, prognosis prediction, and identifying novel treatment targets. Following the publication of these singular omic analyses across a variety of multi-omic types, several recent review articles discuss this untapped potential. For example, the potential benefits of integrative analysis for rare neurological diseases,[13] Dermatomyositis / Polymyositis,[14] liver cancer[15] and inborn errors of metabolism.[16] This review will identify studies were more than one omic term has been employed in studies of rare disease.

A major challenge facing researchers is interpretation of the large amounts of data generated from next generation approaches. In contrast to common complex diseases, rare disease researchers are also faced with the challenge of generating reliable, high quality data, such as a randomised controlled trial, when they often have an extremely small sample size. To tackle this, international collaboration of research is crucial to include participants of a rare disease who may live in different countries, or even continents. Additionally, there is a need to develop a standardised reporting structure for rare disease omic analysis, such as that which exists for genome wide association studies.[17]

This scoping review will highlight important gaps in multi-omic rare disease research and will summarise elements of good practice for rare disease omic analyses, providing evidence for more robust, larger-scale, collaborative studies.

Review aim and objectives.

This scoping review will aim to systematically summarise what research has been conducted into multi-omics and rare disease by:

- Evaluating what primary research studies exist pertaining to multi-omics and rare disease and which type of omic analysis was undertaken.

For peer review only - http://bmjopen.bmj.com/site/about/guidelines.xhtml

- Highlighting research outcomes with implications for rare disease diagnosis, treatment or improved understanding of disease mechanisms.

METHODS

This scoping review protocol was designed using the Joanna Briggs Institute Reviewers Manual 2015 – methodology for scoping reviews,[18] and our completed review will follow the PRISMA Extension for Scoping Reviews (PRISMA-ScR) guidelines.[19]

Eligibility criteria

The inclusion criteria were developed using the PCC (population, concept, context) framework of the Joanna Briggs Institute (JBI) scoping review methodology, with a deliberately wide scope to capture as many relevant research studies as possible. The "population" of this scoping review will be rare diseases meeting the European definition of less than 1 in 2,000.[2] Participants may be rare disease patients of all ages, ethnicity and gender. The "concept" of the review is multi-omics and therefore must include at least two types of omic analysis. The most common omic terms we would expect to see are epigenomics, genomics, metabolomics, phenomics, proteomics and transcriptomics, however any omic term may be included. A comprehensive list of omic terms is available for reference on www.omics.org.[7] The review "context" will be within a primary research study written in English and published prior to the 30th December 2018 and as such other reviews of the literature will not be included in the final synthesis but may be helpful for identification of further relevant studies through reverse screening and forward citation searching.

Information sources and search terms

Articles will be identified primarily by searching the electronic databases MEDLINE via Ovid, EMBASE via Ovid, PubMed, Web of Science, Scopus, and Google Scholar. This large selection of databases will be included in the strategy to perform as wide a search as possible. Tables S1 and S2 of the supplementary files illustrate example search terms for MEDLINE and Embase, which will be adapted for the other databases. As our search terms are limited to papers which directly define the condition as a "rare disease", we have included Google Scholar to enable 'full text

For peer review only - http://bmjopen.bmj.com/site/about/guidelines.xhtml

searching', in order to help address this limitation of our review. These terms were devised based on the PCC framework discussed above and discussed with a faculty librarian. In addition to traditional searching, reverse citation screening of the reference lists of relevant articles (i.e. including the key terms such as multi-omics and rare disease) and forward citations (articles which have cited the identified papers) will be searched for using the Web of Science Cited Reference Search tool.

Selection of studies, data extraction and analysis

The screening process to select studies will begin with compilation of articles identified from the several databases using reference management software (EndNote X8) and subsequent auto-removal of duplicates followed by additional manual checking and removal of further duplicates missed by the software. Title, keyword and abstract screening will identify any studies which noticeably do not meet the study inclusion criteria, i.e. no discussion of multi-omics or singularly focusing on a common disease. Following this, any papers which initially appear to meet the inclusion criteria will be screened *via* reading the full text of the article. Data will then be extracted from the articles which are selected to be included in the review using a customised data extraction form, (Table S3), to include information about study design, participant information, experimental methods and statistical analysis. Variables which may impact omic analysis will be highlighted and discussed where known, including participant age, co-morbidities, gender, ethnicity and type of sample collected. This information will be summarised in a table. The screening and data extraction will be performed in duplicate by two independent personnel to ensure reproducibility, and any discrepancies will be considered by third reviewer. A narrative synthesis of the results of this review will be presented.

Patient and Public Involvement

There was no public or patient involvement in the making of this protocol and will be none in the review, as we are using secondary data from publicly available resources. However, this review will form part of a wider patient-centred research strategy; a multiomic approach to improving rare disease diagnosis within the *100,000 Genomes Project*, as part of the Northern Ireland Genomic Medicine Centre and other genomic medicines centres across the United Kingdom. Collaborative patient-researcher conferences highlighted the need to prioritise improving diagnostic yield, such as the

For peer review only - http://bmjopen.bmj.com/site/about/guidelines.xhtml

'All-Ireland Rare Disease Day Conference' in March 2018 and an '*Advancing registry and information resources for rare diseases'* event in January 2018.

ETHICS AND DISSEMINATION

As this is a review of previously published data and no primary data will be collected, formal ethical committee approval is not required for this scoping review. This scoping review will highlight primary studies that have been conducted for multi-omic analysis of rare disease and discuss the quality of any such research. Multi-omic analysis of rare diseases has important implications for diagnostic/prognostic biomarker development, identifying new treatments, and improving our understanding of the diseases themselves. Subsequently, any gaps in the literature which are identified will be presented at conferences, research group meetings and submitted for publication in a peer reviewed journal. This review will also form part of a chapter in KK's PhD thesis. This review may help guide future rare disease research.

AUTHOR CONTRIBUTIONS

AJ McK, HMcA conceptualised the review protocol, KK drafted the review protocol and all authors were involved in preparing and agreeing the final protocol manuscript.

ACKNOWLEDGMENTS

The authors would like to thank Richard Fallis, the School of Medicine, Dentistry and Biomedical Sciences Faculty librarian, for his consultation when developing the search terms.

FUNDING STATEMENT

KK is supported by a Department for the Economy Co-operative Awards in Science and Technology (DfE-CAST) studentship award. Funding support was provided by the Medical Research Council – Northern Ireland Executive support of the Northern Ireland Genomic Medicine Centre though Belfast Health and Social Care Trust.

For peer review only - http://bmjopen.bmj.com/site/about/guidelines.xhtml

COMPETING INTEREST STATEMENT

No competing interests to declare.

REFERENCES

1. RARE DISEASES - A major unmet medical need. Luxembourg: European Commission 2017.

2. Global Genes. RARE Diseases: Facts and Statistics [Available from: https://globalgenes.org/rare-diseases-facts-statistics/ accessed 09/11/2018.

3. EURODIS. About Rare Diseases [Available from: https://www.eurordis.org/about-rare-diseases accessed 15/03/2018.

4. What is a rare disease? : Genetic Alliance UK; 2016 [Available from: http://www.raredisease.org.uk/what-is-a-rare-disease/ accessed 16/04/2018.

5. Boycott KM, Vanstone MR, Bulman DE, et al. Rare-disease genetics in the era of next-generation sequencing: discovery to translation. *Nature reviews Genetics* 2013;14(10):681-91. doi: 10.1038/nrg3555 [published Online First: 2013/09/04]

6. Hasin Y, Seldin M, Lusis A. Multi-omics approaches to disease. *Genome Biology* 2017;18(1):83. doi: 10.1186/s13059-017-1215-1

7. Alphabetically ordered list of omes and omics 2016 [Available from: http://omics.org/index.php/Alphabetically_ordered_list_of_omes_and_omics accessed 16/03/2018.

8. Sallustio F, Serino G, Cox SN, et al. Aberrantly methylated DNA regions lead to low activation of CD4+ T-cells in IgA nephropathy. *Clinical science (London, England : 1979)* 2016;130(9):733-46. doi: https://dx.doi.org/10.1042/CS20150711

9. Woo YM, Bae J-B, Oh Y-H, et al. Genome-wide methylation profiling of ADPKD identified epigenetically regulated genes associated with renal cyst development. *Human genetics* 2014;133(3):281-97. doi: https://dx.doi.org/10.1007/s00439-013-1378-0

For peer review only - http://bmjopen.bmj.com/site/about/guidelines.xhtml

10. Khuc E, Bainer R, Wolf M, et al. Comprehensive characterization of DNA methylation changes in Fuchs endothelial corneal dystrophy. *PLoS One* 2017;12(4):e0175112. doi: 10.1371/journal.pone.0175112 [published Online First: 2017/04/07]

11. Farinelli P, Perera A, Arango-Gonzalez B, et al. DNA methylation and differential gene regulation in photoreceptor cell death. *Cell Death Dis* 2014;5:e1558. doi: 10.1038/cddis.2014.512 [published Online First: 2014/12/06]

12. Farkas MH, Au ED, Sousa ME, et al. RNA-Seq: Improving Our Understanding of Retinal Biology and Disease. *Cold Spring Harbor Perspectives in Medicine* 2015;5(9):a017152. doi: 10.1101/cshperspect.a017152

13. Crowther LM, Poms M, Plecko B. Multiomics tools for the diagnosis and treatment of rare neurological disease. *Journal of Inherited Metabolic Disease* 2018;41(3):425-34. doi: 10.1007/s10545-018-0154-7

14. Gao S, Luo H, Zhang H, et al. Using multi-omics methods to understand dermatomyositis/polymyositis. *Autoimmunity reviews* 2017;16(10):1044-48. doi: 10.1016/j.autrev.2017.07.021 [published Online First: 2017/08/06]

15. Lin S, Yin YA, Jiang X, et al. Multi-OMICs and Genome Editing Perspectives on Liver Cancer Signaling Networks. *BioMed research international* 2016;2016:6186281. doi: 10.1155/2016/6186281 [published Online First: 2016/07/13]

16. Argmann CA, Houten SM, Zhu J, et al. A Next Generation Multiscale View of Inborn Errors of Metabolism. *Cell metabolism* 2016;23(1):13-26. doi: 10.1016/j.cmet.2015.11.012 [published Online First: 2015/12/30]

17. Little J, Higgins JP, Ioannidis JP, et al. STrengthening the REporting of Genetic Association Studies (STREGA)--an extension of the STROBE statement. *Genetic epidemiology* 2009;33(7):581-98. doi: 10.1002/gepi.20410 [published Online First: 2009/03/12]

18. Joanna Briggs Institute Reviewers' Manual: 2015 edition/supplement. Australia: The Joanna Briggs Institute, 2015.

19. Tricco AC, Lillie E, Zarin W, et al. PRISMA Extension for Scoping Reviews (PRISMA-ScR): Checklist and Explanation. *Ann Intern Med* 2018;169(7):467-73. doi: 10.7326/m18-0850 [published Online First: 2018/09/05]

For peer review only - http://bmjopen.bmj.com/site/about/guidelines.xhtml

Supplementary materials and methods.

Protocol for a scoping review of multi-omic analysis for rare diseases.

Ms Katie Kerr, Dr Helen McAneney, Dr Amy Jayne McKnight.

Table S1 - Search terms for Ovid MEDLINE(R) ALL.

#	**Searches**
1	Rare Diseases/
2	("rare disease*" or "rare cancer*" or "rare syndrome*" or "rare disorder*").mp.
3	multi?omic*.mp.
4	genomics/ or epigenomics/ or proteomics/ or transcriptomics/ or metabolomics/
5	"integrat* omic*".mp. [mp=title, abstract, original title, name of substance word, subject heading word, floating sub-heading word, keyword heading word, protocol supplementary concept word, rare disease supplementary concept word, unique identifier, synonyms]
6	"integrat* analys*".mp. [mp=title, abstract, original title, name of substance word, subject heading word, floating sub-heading word, keyword heading word, protocol supplementary concept word, rare disease supplementary concept word, unique identifier, synonyms]
7	1 or 2
8	3 or 4 or 5 or 6
9	7 and 8

For peer review only

For peer review only - http://bmjopen.bmj.com/site/about/guidelines.xhtml

Table S2 – Search terms for Embase via Ovid.

#	**Searches**
1	rare disease/
2	("rare disease*" or "rare cancer*" or "rare syndrome*" or "rare disorder*").mp.
3	multi?omic*.mp.
4	genomics/
5	epigenetics/
6	proteomics/
7	transcriptomics/
8	metabolomics/
9	epigenomic*.mp. [mp=title, abstract, heading word, drug trade name, original title, device manufacturer, drug manufacturer, device trade name, keyword, floating subheading word, candidate term word]
10	"integrat* omic*".mp.
11	"integrat* analys*".mp.
12	1 or 2
13	3 or 4 or 5 or 6 or 7 or 8 or 9 or 10 or 11
14	11 and 12

For peer review only - http://bmjopen.bmj.com/site/about/guidelines.xhtml

Table S3. Template for customised data extraction form.

First author and publication year	Title and digital object identifier	Reference list number	Research objective	Phenotypes reported	Experimental controls used	Statistical analysis	Study design and participant information	Key results

For peer review only - http://bmjopen.bmj.com/site/about/guidelines.xhtml

PRISMA-P (Preferred Reporting Items for Systematic review and Meta-Analysis Protocols) 2015 checklist: recommended items to address in a systematic review protocol*

Section and topic	Item No	Checklist item	Line Number
**ADMINISTRATIVE INFORMATION**			
Title:			N/A, Scoping Review, Line 2.
Identification	1a	Identify the report as a protocol of a systematic review	
Update	1b	If the protocol is for an update of a previous systematic review, identify as such	N/A
Registration	2	If registered, provide the name of the registry (such as PROSPERO) and registration number	N/A
Authors:			4 – 13
Contact	3a	Provide name, institutional affiliation, e-mail address of all protocol authors; provide physical mailing address of corresponding author	196-198
Contributions	3b	Describe contributions of protocol authors and identify the guarantor of the review	
Amendments	4	If the protocol represents an amendment of a previously completed or published protocol, identify as such and list changes; otherwise, state plan for documenting important protocol amendments	N/A
Support:			
Sources	5a	Indicate sources of financial or other support for the review	205 – 209<br>205 – 209<br>N/A
Sponsor	5b	Provide name for the review funder and/or sponsor	
Role of sponsor or funder	5c	Describe roles of funder(s), sponsor(s), and/or institution(s), if any, in developing the protocol	
**INTRODUCTION**			
Rationale	6	Describe the rationale for the review in the context of what is already known	65 – 112
Objectives	7	Provide an explicit statement of the question(s) the review will address with reference to participants, interventions, comparators, and outcomes (PICO)	113 - 119
**METHODS**			
Eligibility criteria	8	Specify the study characteristics (such as PICO, study design, setting, time frame) and report characteristics (such as years considered, language, publication status) to be used as criteria for eligibility for the review	126 - 140

For peer review only

For peer review only - http://bmjopen.bmj.com/site/about/guidelines.xhtml

Information sources	9	Describe all intended information sources (such as electronic databases, contact with study authors, trial registers or other grey literature sources) with planned dates of coverage	141 - 154
Search strategy	10	Present draft of search strategy to be used for at least one electronic database, including planned limits, such that it could be repeated	155 - 172
Study records:			
Data management	11a	Describe the mechanism(s) that will be used to manage records and data throughout the review	157
Selection process	11b	State the process that will be used for selecting studies (such as two independent reviewers) through each phase of the review (that is, screening, eligibility and inclusion in meta-analysis)	156 – 163
Data collection process	11c	Describe planned method of extracting data from reports (such as piloting forms, done independently, in duplicate), any processes for obtaining and confirming data from investigators	163 – 165
Data items	12	List and define all variables for which data will be sought (such as PICO items, funding sources), any pre-planned data assumptions and simplifications	127 – 140
Outcomes and prioritization	13	List and define all outcomes for which data will be sought, including prioritization of main and additional outcomes, with rationale	163 – 166
Risk of bias in individual studies	14	Describe anticipated methods for assessing risk of bias of individual studies, including whether this will be done at the outcome or study level, or both; state how this information will be used in data synthesis	N/A
Data synthesis	15a	Describe criteria under which study data will be quantitatively synthesised	N/A
	15b	If data are appropriate for quantitative synthesis, describe planned summary measures, methods of handling data and methods of combining data from studies, including any planned exploration of consistency (such as I^2, Kendall's τ)	N/A<br>N/A
	15c	Describe any proposed additional analyses (such as sensitivity or subgroup analyses, meta-regression)	
	15d	If quantitative synthesis is not appropriate, describe the type of summary planned	171 - 172
Meta-bias(es)	16	Specify any planned assessment of meta-bias(es) (such as publication bias across studies, selective reporting within studies)	N/A
Confidence in cumulative evidence	17	Describe how the strength of the body of evidence will be assessed (such as GRADE)	N/A

For peer review only

*** It is strongly recommended that this checklist be read in conjunction with the PRISMA-P Explanation and Elaboration (cite when available) for important clarification on the items. Amendments to a review protocol should be tracked and dated. The copyright for PRISMA-P (including checklist) is held by the PRISMA-P Group and is distributed under a Creative Commons Attribution Licence 4.0.**

From: Shamseer L, Moher D, Clarke M, Ghersi D, Liberati A, Petticrew M, Shekelle P, Stewart L, PRISMA-P Group. Preferred reporting items for systematic review and meta-analysis protocols (PRISMA-P) 2015: elaboration and explanation. BMJ. 2015 Jan 2;349(jan02 1):g7647.
